# Clinical experience with tigecycline in the treatment of hospital-acquired pneumonia caused by multidrug resistant *Acinetobacter baumannii*

**DOI:** 10.1186/s40360-019-0300-3

**Published:** 2019-04-25

**Authors:** Yangang Zhou, Xumin Chen, Ping Xu, Yan Zhu, Kuangguo Wang, Daxiong Xiang, Feng Wang, Hoan Linh Banh

**Affiliations:** 1 Department of Pharmacy, the Second Xiangya Hospital, Central South University, No.139 Middle Renmin Road, Changsha, Hunan 410011 People’s Republic of China; 2 Institute of Clinical Pharmacy, Central South University, Changsha, People’s Republic of China; 3 The Traditional Chinese Medicine hospital of Longhui, Changsha, Hunan People’s Republic of China; 4 Department of Family Medicine, University of Alberta, Edmonton, Canada

**Keywords:** Tigecycline, Multi-drug resistance, *Acinetobacter baumannii*, Hospital-acquired pneumonia

## Abstract

**Background:**

Tigecycline, with broad *in vitro* antibacterial activity, has been widely used off-label for nosocomial pneumonia caused by multi-drug resistant *Acinetobacter baumannii* (MDRAB). However, many concerns have been raised about the efficacy of tigecycline treatment as the inconsistent results from previous clinical studies.

**Methods:**

This retrospective study evaluated the outcome of adult patients with monomicrobial MDRAB nosocomial pneumonia treated with tigecycline between 2015 and 2017. Results.

A total of 77 patients was eligible for this study, and the overall clinical success and 30-day survival rates were 70.03 and 70.13%, respectively, however, the microbiological eradication rate was relatively low (48%). Multivariate analysis indicated that shorter duration of tigecycline use associated with increased clinical failure, whereas higher CURB65 scores, mechanical ventilation and tigecycline resistant to MDRAB have significant association with 30-day mortality.

**Conclusions:**

Our results suggest that tigecycline is one of the potential choices for the treatment of hospital-acquired pneumonia caused by MDRAB, especially with a MIC≤2 mg/L. In addition, a longer duration of tigecycline treatment may be required to insure better clinical outcomes.

## Backgrounds

Pneumonia caused by multidrug-resistant *Acinetobacter baumannii* (MDRAB) is a challenge in nosocomial infection as it has become resistant to most antibiotics, including penicillins, cephalosporins, aztreonam, fluoroquinolones, aminoglycosides and even carbapenems, resulting in limited antibiotic options for treatment [1, 2].

Tigecycline, the first member of the glycylcycline class of antimicrobial agents, has been approved by the Food and Drug Administration (FDA) for the treatment of complicated skin and skin-structure infections, complicated intra-abdominal infections and community-acquired pneumonia [3]. Because of its expanded spectrum of in vitro antibacterial activity, and high sensitivity to MDRAB, tigecycline has been widely used off-label for nosocomial pneumonia caused by MDRAB [4, 5].

According to previous studies, tigecyclineis no better than standard antimicrobial agents for the treatment of various infections caused by MDRAB, and mortality from all causes was even higher in the tigecycline group [6–9]. However, since most of these studies focused on the multisite infections with more than one pathogen, it is difficult to establish the effect of tigecycline as a single agent on MDRAB with so many confounding factors. As a result, it is unclear whether tigecycline is an effective option for treating pneumonia caused by MDRAB. Therefore, we conducted a retrospective study with monomicrobial MDRAB pneumonia to assess the effectiveness of tigecycline for the treatment of MDRAB nosocomial pneumonia, and to identify the predictors of treatment success.

## Methods

### Study design, subjects and treatments

This was a retrospective study performed in the second Xiangya Hospital of Central South University (Changsha, China), a tertiary-care teaching hospital with more than 3000 beds, between January 2015 and December 2017. The study received approval from the Second Xiangya Hospital of Central South University Ethics Committee. All hospitalized patients age ≥ 18 years who received tigecycline for the treatment of hospital-acquired pneumonia (HAP) involving monomicrobial MDRAB were included in this study. Tigecycline treatment was at least 5 days, with a 100 mg loading dose followed by 50 mg administered intravenously every 12 h. Data included demographic characteristics, medical history, clinical and laboratory findings, diagnostic imaging and type of treatment and outcome were extracted from the electronic patient medical records. The primary study outcomes were clinical and microbiologic success rates, and we also investigated the factors associated with clinical failure and mortality.

### Definition

Hospital-acquired pneumonia (HAP) was defined as pneumonia that occurred 48 h or more after admission. Pneumonia was diagnosed according to the American Thoracic Society Guidelines 2005, which consists of a new or progressive infiltrate on chest X-ray with two or more of the following clinical characteristics: new onset of fever (≥38 °C) or hypothermia (< 35.5 °C), leukocytosis (leukocyte count> 10,000 cells/mm^3^) or leukopenia (leukocyte count< 4000 cells/mm3), oxygen desaturation, and positive purulent sputum [10]. Monomicrobial MDRAB pneumonia was defined as a positive sputum or tracheal aspirate culture of only MDRAB from 1 week before up to 1 week after the initiation of first dose of tigecycline, and there was no other infection at the start of the treatment. Severity of pneumonia was based on a CURB-65 score which was recorded within 48 h of the administration of first dose of tigecycline. All cause 30-day mortality was defined as death during 30 days of treatment with tigecycline. Clinical resolution of pneumonia at the end of treatment was defined as improvement of the subsequent chest X-ray, or partial or complete resolution of signs and symptoms of infection and improvement or resolution of laboratory tests such as white blood cell count (decrease to normal), C-reactive protein (CRP) (decrease to normal or by≥30%), and procalcitonin (PCT) (decrease to normal or by≥80%) [11]. Delayed tigecycline treatment was defined as 3 days delay of tigecycline use after the detection of airway MDRAB isolates [12].

### Microbiology

The antibiotic susceptibility profiling of isolates had been performed (except tigecycline and cefoperazone sulbactam) using a BD Phoenix-100 automated microbiology system (Diagnostic Systems, Sparks, MD) [13]. The results were interpreted according to the breakpoints suggested by the Clinical and Laboratory Standards Institute (CLSI) 2016. Susceptibility to tigecycline was determined using disk diffusion method Standard of antibiotics Susceptibility Test (Kirby-Bauer Method), and the diameter of inhibition zone≥16 mm, 13-15 mm, ≤12 mm interpreted as susceptible, intermediately resistant and resistant, respectively. MDRAB was defined as *A. baumonnii* resistance to at least three of the following classes of antibiotics: aminoglycosides, antipseudomonal penicillins, carbapenems, cephalosporins and quinolones. Microbiological eradication was defined as the absence of MDRAB from all subsequent respiratory cultures. Bacteremia was defined as one or more positive blood culture for *Acinetobacter baumannii* during tigecycline treatment.

### Statistical methods

All statistical analyses were performed using the Statistical Package for the Social Sciences for Windows (Version 18.0; SPSS Inc., Chicago, IL, USA). Categorical variables were compared using chi-square test. Continuous variables were tested for normality of distributions by Kolmogorove-Smirnov test, and then compared by the Mann-Whitney U test. Odd ratios (ORs) and 95% confidence interval (CI) were calculated. Variables with a *p* value < 0.05 in univariate analysis were included in a logistic regression model for multivariate analysis. All tests were two-tailed, and a *p* value < 0.05 was considered statistically significant in multivariate analysis.

## Result

### Patients, demography and concomitant diseases

**Fig. 1:**
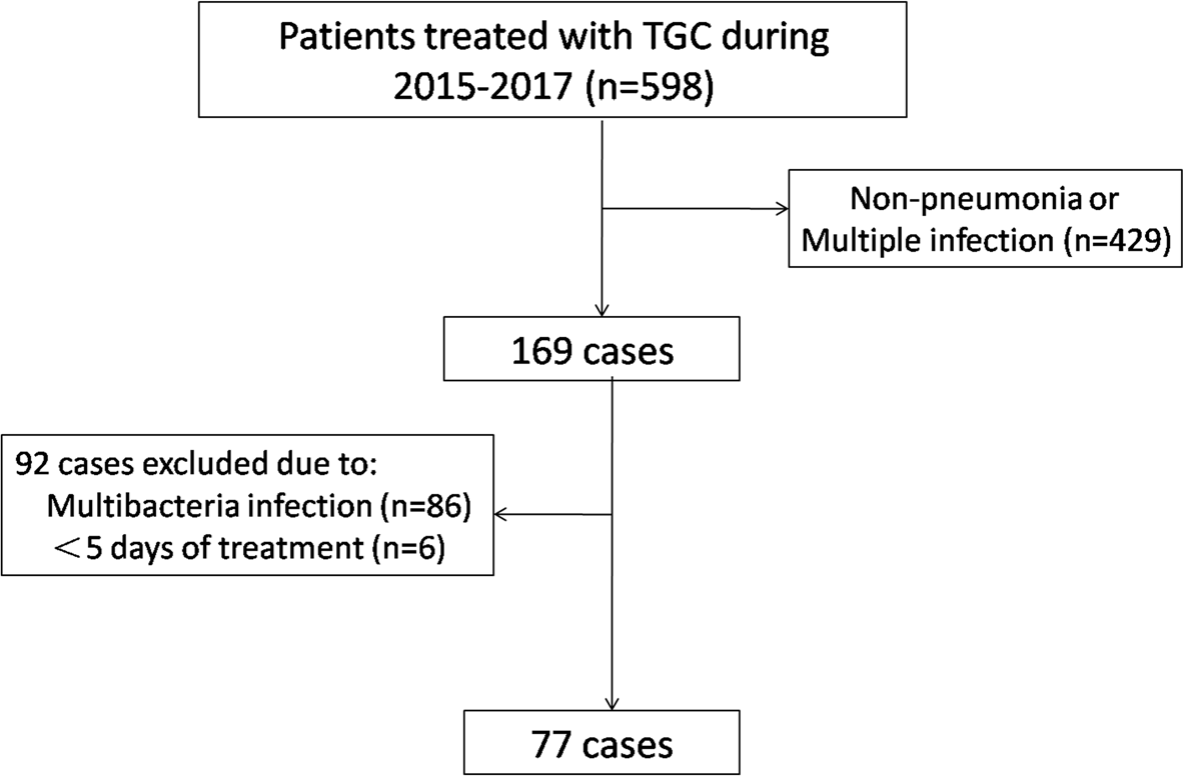
Allocation of MDR/XDRAB pneumonia patients included. MDR/XDRAB = multidrug-resistant and extensively drug-resistant Acinetobacter baumannii; TGC = tigecycline.

There were 598 patients received tigecycline between 2015 and 2017. A total of 77 patients with monomicrobial MDRAB were identified (Fig. 1), patient demographics and clinical features are summarized in Table 1. The mean age was 62.1 years and 52 (67.53%) were male. The major comorbidities were chronic obstructive pulmonary disease (COPD) (31.17%) and hypertension (22.08%) and 16 patients had surgery (20.78%).

**Table 1:** Characteristics of the 77 patients of tigecycline-treated pneumonia involving multidrugresistant *Acinetobacter baumannii* (MDRAB)

Characteristics	values
Demographic parameters	
Age, mean ± SD, years	62.1 ± 17.0
Male/Female	52/25
Comorbidities	
Hepatic dysfunction	2 (2.6%)
Renal insufficiency	7 (9.1%)
Chronic pulmonary disease	24 (31.2%)
Heart disease	14 (18.2%)
Hypertension	17 (22.1%)
Diabetes mellitus	6 (7.8%)
Immune compromise	5 (6.5%)
multiple organ failure	3 (3.9%)
Malignancy	8 (10.4%)
Surgery	16 (20.8%)
Clinical conditions	
CURB65 score, mean ± SD	2.0 ± 1.0
Mechanical ventilation	48 (62.3%)
Susceptibility tests of initial airway MDRAB isolates	
With sensitive to tigecycline	42 (54.5%)
With sensitive to sulbactam	7 (9.1%)
Tigecycline treatment	
Duration, mean ± SD, days	11.69 ± 6.11
Combination therapy	71 (92.2%)
With sulbactam	64 (90.1%)
With carbapenems	4 (5.6%)
With amikacin	1 (1.4%)
With fluoroquinolones	4 (5.6%)
Delayed tigecycline treatment	21 (27.3%)

Abbreviations: *SD* standard deviation

### Clinical characteristics of infection and treatment

The mean CURB65 score was 2.02. CURB65 severity score for pneumonia severity is summarized in Table 2 [14]. Almost half of the patients, 48 (62.34%), were on mechanical ventilation during tigecycline treatment. Almost all of the cultures with only MDRAB isolated were from airway specimens with only 7 of cultures are from blood samples. Among the 77 MDRAB isolates, 52 (67.53%) and 20 (25.97%) were resistant or intermediately resistant to sulbactam, and were all resistant to carbapenems, fluoroquinolones and amikacin. Only 6 isolates (7.79%) were resistant, and 29 (37.66%) were intermediate resistant to tigecycline. No resistance to colistin was found. The mean duration of tigecycline use was 11.69 days, and treatment was discontinued in 3 patients on day 5 of treatment due to diarrheal and the elevator of serum total bilirubin level. There were 71 patients (92.21%) received combination therapy with tigecycline. Cefoperazone sulbactam was the most common co-administered agents (64/71, 90.14%), followed by carbapenems and fluoroquinolones (4/71, 5.63%); 21 patients (27.27%) had delayed tigecycline treatment (Table 1).

**Table 2:** CURB65 Severity for Pneumonia

Score	Risk	Disposition
0, or 1	1.5% mortality	Outpatient care
2	9.2% mortality	Inpatient vs observation admission
**≥** 3	22% mortality	Inpatient admission with consideration for ICU admission with score of 4 or 5

### Microbiological outcomes

There were 50 patients that had subsequent follow-up respiratory tract cultures after the tigecycline treatment, and 24(48%) of them had airway eradication from MDRAB.

### Clinical outcomes

The all cause 30-day mortality rate was (29.87%). Only 58 patients had follow-up chest radiographs after tigecycline treatment with 21(36.21%) patients showed improvement and 57 (70.03%) patients had clinical resolution of pneumonia.

### Risk factors for clinical resolution of tigecycline treatment for MDRAB pneumonia

In univariate analysis, patients without clinical resolution of pneumonia had higher CURB65 scores, higher rates of ventilator associated pneumonia (VAP) and bacteremia caused by MDRAB, delayed tigecycline treatment, shorter duration of tigecycline use and lower ratio of airway eradication of MDRAB when comparing to the patients with clinical resolution (*p*< 0.05) (Table 3). Variables with a *p* value< 0.05 were included in multivariate analysis, and the independent predictor for failure of clinical resolution was the shorter duration of tigecycline use (Table 4).

**Table 3:** Univariate analysis of the predictors for failure in clinical resolution (CR)

Variables	With CR^a^ *n* = 57	Without CR^a^ *n* = 20	OR (95% CI)	*p*
Demographic parameters				
Age, year	59.4 (18.9)	63.09 (16.3)		0.464
Female gender	16 (28.1%)	9 (45.0%)	0.48 (0.17–1.37)	0.164
Comorbidities				
Hepatic dysfunction	2 (3.5%)	0 (0.0%)	0.73 (0.64–0.84)	1.000
Renal insufficiency	6 (10.5%)	1 (5.0%)	2.24 (0.25–19.80)	0.669
Chronic pulmonary disease	14 (24.6%)	7 (35.0%)	0.60 (0.20–1.82)	0.367
Heart disease	11 (19.3%)	2 (10.0%)	2.15 (0.43–10.68)	0.495
Diabetes mellitus	3 (5.3%)	1 (5.0%)	1.06 (0.10–10.77)	1.000
Immune compromise	3 (5.3%)	2 (10.0%)	0.50 (0.08–3.23)	0.600
Malignancy	5 (8.8%)	2 (10.0%)	0.86 (0.15–4.86)	1.000
Hypoproteinemia	1 (1.8%)	1 (5.0%)	0.34 (0.20–5.69)	0.455
Surgery	11 (19.3%)	5 (25.0%)	0.72 (0.22–2.34)	0.749
Clinical conditions				
CURB65 score, mean ± SD	2.4 (0.6)	1.9 (1.1)		0.006
Mechanical ventilation	31 (64.6%)	17 (85.0%)	0.21 (0.06–0.80)	0.015
Albumin	31.7 (5.7)	32.2 (4.0)		0.512
Microbiology				
Bacteremia	2 (3.5%)	5 (25.0%)	0.11 (0.02–0.62)	0.011
Tigecycline resistance	33 (57.9%)	9 (45.0%)	1.68 (0.60–4.69)	0.319
Airway eradication of MDRAB	21 (51.2%)	3 (18.8%)	4.55 (1.13–18.39)	0.026
Tigecycline treatment				
Duration, days	8.4 (3.7)	12.9 (6.4)		0.001
Combination therapy				
With sulbactam	49 (86.0%)	15 (75.0%)	2.04 (0.58–7.19)	0.304
With carbapenems	3 (5.3%)	1 (5.0%)	1.06 (0.10–10.77)	1.000
With amikacin	2 (3.6%)	1 (5.0%)	0.70 (0.06–8.21)	1.000
With fluoroquinolones	4 (7.0%)	0 (0.0%)	0.73 (0.63–0.84)	0.568
Delayed tigecycline treatment	19 (33.3%)	2 (10.0%)	4.50 (0.86–13.31)	0.044

Abbreviations: *OR* odd ratio, *CI* confidence interval, *MDRAB* multidrugresistik Acinetobacter baumannii

^a^Categorical data are no.(%) of subject, continuous data are expressed as mean (standard deviation)

**Table 4:** Multivariate analysis of the predictors for failure in clinical resolution

Variables	Adjusted OR (95% CI)	*p*
CURB65 score	0.65 (0.35–1.23)	0.187
Mechanical ventilation	0.33 (0.07–1.59)	0.165
Bacteremia	0.21 (0.02–2.46)	0.213
Airway eradication of MDRAB	1.45 (0.63–3.34)	0.382
Duration of Tigecycline treatment	1.23 (1.02–1.48)	0.031
Delayed tigecycline treatment	3.60 (0.62–20.90)	0.153

Abbreviations: *OR* odd ratio, *CI* confidence interval, *MDRAB* multidrugresistant Acinetobacter baumannii

^a^Categorical data are n (%) of subject, continuous data are expressed as mean (standard deviation)

### Risk factors for the 30-day mortality

In univariate analysis, patients with 30-day mortality had higher CURB65 scores, higher resistance rate to tigecycline, VAP and bacteremia caused by MDRAB, and shorter duration of tigecycline use when comparing to the survival patients (*p*< 0.05) (Table 5). Variables with a *p* value< 0.05 were included in multivariate analysis, and the independent predictors for 30-day mortality were higher CURB65 score, VAP and tigecycline resistance (Table 6).

**Table 5:** Univariate analysis of the predictors for the 30-day mortality

Variables	Death^a^ *n* = 23	Survival^a^ *n* = 54	OR (95% CI)	*p*
Demographic parameters				
Age, year	63.0 (18.5)	61.8 (16.5)		0.644
Female gender	9 (39.1%)	16 (29.6%)	1.53 (0.55–4.24)	0.415
Comorbidities				
Hepatic dysfunction	0 (0.0%)	2 (3.7%)	0.69 (0.60–0.81)	1.000
Renal insufficiency	1 (4.3%)	6 (11.1%)	0.36 (0.41–3.21)	0.667
Chronic pulmonary disease	6 (26.1%)	15 (27.8%)	0.92 (0.30–2.78)	0.879
Heart disease	2 (8.7%)	11 (20.4%)	0.37 (0.08–1.83)	0.323
Diabetes mellitus	2 (8.7%)	2 (3.7%)	2.48 (0.33–18.75)	0.578
Immune compromise	2 (8.7%)	3 (5.6%)	1.62 (0.25–10.40)	0.632
Malignancy	2 (8.7%)	5 (9.3%)	0.93 (0.17–5.20)	1.000
Hypoproteinemia	0 (0.0%)	2 (3.7%)	0.69 (0.60–0.81)	1.000
Surgery	5 (21.7%)	11 (20.4%)	1.09 (0.33–3.58)	1.000
Clinical conditions				
CURB65 score, mean ± SD	2.7 (1.1)	1.7 (0.8)		0.001
Mechanical ventilation	21 (91.3%)	27 (50.0%)	10.5 (2.24–49.24)	0.001
Albumin	32.2 (4.9)	32.1 (4.3)		0.916
Microbiology				
Bacteremia	5 (21.7%)	2 (3.7%)	7.22 (1.29–40.54)	0.022
Tigecycline resistance	16 (69.6%)	19 (35.2%)	0.24 (0.08–0.68)	0.006
Airway eradication of MDRAB	5 (27.8%)	19 (48.7%)	0.40 (0.12–1.35)	0.137
Tigecycline treatment				
Duration, days	10.0 (6.6)	12.4 (5.8)		0.014
Combination therapy				
With sulbactam	19 (82.6%)	45 (83.3%)	0.95 (0.26–3.47)	1.000
With carbapenems	1 (4.3%)	3 (5.6%)	0.77 (0.08–7.84)	1.000
With amikacin	1 (4.3%)	2 (3.8%)	1.16 (0.10–13.46)	1.000
With fluoroquinolones	1 (4.3%)	3 (5.6%)	0.77 (0.08–7.84)	1.000
Delayed tigecycline treatment	6 (26.1%)	15 (27.8%)	0.92 (0.30–2.77)	0.879

Abbreviations: *OR* odd ratio, *CI* confidence interval, *MDRAB* multi-drug-resistant Acinetobacter baumannii

^a^Categorical data are n (%) of subject, continuous data are expressed as mean (standard deviation)

**Table 6:** Multivariate analysis of the predictors for the 30-day mortality

Variables	Adjusted OR (95% CI)	*p*
CURB65 score	3.18 (1.44–6.99)	0.004
Mechanical ventilation	6.38 (1.16–35.23)	0.034
Bacteremia	21.00 (0.99–443.40)	0.050
Duration of Tigecycline treatment	0.97 (0.87–1.08)	0.567
Tigecycline resistance	0.20 (0.05–0.78)	0.021

Abbreviations: *OR* odd ratio, *CI* confidence interval, *MDRAB* multidrugresistant Acinetobacter baumannii

## Discussion

This study evaluated the outcomes of 77 patients with monomicrobial MDRAB nosocomial pneumonia treated with tigecycline. The overall clinical resolution and 30-day survival rates were 70.0 and 70.1%, respectively. The microbiological eradication rate was 48.0%. Based on previous studies, the clinical resolution of tigecycline treatment was between 45.2 and 84.0% [15–17], and the difference between studies may be due to the severity of illness, since the clinical success rate were relatively lower in intensive care patients who had higher APACHEII scores. Similar to previous studies, positive microbiological response rates were lower when compared with successful clinical outcome in this study. The concerns were the inconsistency of clinical success and microbiologic response in previous studies may not be attributed to tigecycline, rather it is due to misdiagnosis. Curcio [18] et al. found that the non-bronchoalveolar lavage method for microbiology sampling predicts poor clinical outcomes, which means that using bronchoalveolar lavage (BAL) may result in higher accuracy in identifying microbiologic organisms and reduce the risk of misdiagnosis. This study only included cultures positive for monomicrobial MDRAB, and among the patients with follow-up sputum cultures, the clinical success rate was significantly higher in the group of microbiological eradication (21/24, 87.5% vs 20/33, 60.6%; *p* = 0.026). Hence, the lower microbiological response in this study may be due to the strict inclusion criteria compared to previous studies, since only complete eradication of MDRAB was defined as microbiological success. The clinical success was higher as the definition was less strict with both partial and complete resolution of clinical symptoms. This finding has also been shown in other clinical studies evaluating antimicrobials for treatment of hospital-acquired pneumonia (HAP) or ventilator-associated pneumonia (VAP) which made it difficult to directly compare the effect of different antibiotics [19]. Thus, it is essential to standardize terms used in enrolment criteria, endpoints, microbiological and clinical cure in future studies to better understand the results.

To use tigecycline appropriately, predictors for clinical success and 30-day mortality in patients with monomicrobial MDRAB nosocomial pneumonia were investigated in this study. The multivariate analyzes showed that the prolonged tigecycline usage was significantly associated with clinical resolution, whereas higher CURB65 scores, mechanical ventilation and tigecycline resistant to MDRAB have significant association with 30-day mortality, and these results may help to explain previous results. Many studies compared the efficacy of tigecycline in the treatment of MDRAB with other antibiotics, and tigecycline was not recommended as first line treatment for MDRAB because of the higher mortality and lower microbiological success, especially for bacteremia [6–8, 20]. However, further analysis showed that the increased mortality rate of tigecycline is significant higher only among those with MIC>2 mg/L, but not for those with MIC≤2 mg/L. Kim et al. [21] investigated the effectiveness of tigecycline-based versus colistin-based therapy for treatment of MDRAB pneumonia. There was no difference between the groups with respect to clinical outcomes, microbiological success and 30-day mortality, in which most MDRAB isolates showed a tigecycline MIC≤2 mg/L. Hence, the sensitivity of tigecycline might be the key when choosing the regimen against HAP caused by monomicrobial MDRAB. With respect to mortality, MDRAB with MIC>2 mg/L may not be appropriate to treat MDRAB bacteremia with tigecycline 50 mg every 12 h since tigecycline achieve low plasma concentration at this dose. Of note, the present study showed that longer treatment duration may produce better clinical outcomes; this may due to the prolonged time needed for bacteriostatic agent to completely eradicate the bacteria. Since tigecycline is a bacteriostatic agent, it is possible that a longer duration of treatment is required to treat MDRAB.

Many studies investigated the effect of albumin on antibacterial therapy, especially in critical ill patients. The change of albumin level may significantly affect the pharmacokinetics of antibiotics, subsequently lead to altered antibacterial effect [22, 23]. Sujata and Tasbakan [24] found that higher albumin was associated with better clinical and microbiological responses to therapy, the results from this study, however, did not show this relationship, it may because only two patients in the study had hypoalbuminemia. As a result, the difference between two groups was not detected.

For all the MDRAB isolates in this study the non-susceptible rate to tigecycline was 42.9% (33/77) and 81.8% (27/33) were intermediate. High rate of resistant to tigecycline have also been reported previously in Asia. Although the mechanism of tigecycline resistance has not been identified, Park [25] showed that previously receiving carbapenems is an independent risk factor associated with the development of MDRAB resistant to tigecycline. Similarly, most patients in the study (70.1%, 54/77) had previously received carbapenems. Surprisingly, only 8 patients had tigecycline-resistant MDRAB after tigecycline use, and it is consistent with other studies. This study is consistent with previous studies which suggest that previous exposure to a carbapenem increases the risk of *Acinetobacter spp*. resistant to tigecycline [26–28]. There are several limitations in this study. First, it was a single-center study. Although only monomicrobial MDRAB pneumonia was included in the study, the results may not be generalized in other infections and to all settings. Second, this is a retrospective study. The data collection from patient medical records may not be complete.

## Conclusion

Tigecycline is a potential option for the treatment of nosocomial pneumonia infection caused by MDRAB, and to use it appropriately, factors such as the susceptibility of tigecycline to the isolate MDRAB should be considered, as the effect of tigecycline may not be effective when the MIC>2 mg/L. In addition, the predictors for 30-day mortality are high CURB65 scores, higher resistance rate to tigecycline in the hospital, VAP and bacteremia caused by MDRAB, and shorter duration of tigecycline treatment.

## Abbreviations

CI: Confidence interval
CLSI: Clinical and Laboratory Standards Institute
COPD: Chronic obstructive pulmonary disease
CRP: C-reactive protein
HAP: Hospital-acquired pneumonia
MDRAB: multidrug resistent Acinetobacter baumannii
Ors: Odd ratios
PCT: Procalcitonin
VAP: Ventilator associated pneumonia
